# Online public information about advance care planning: An evaluation of UK and international websites

**DOI:** 10.1177/20552076231180438

**Published:** 2023-06-22

**Authors:** Anne Canny, Bruce Mason, Clare Atkins, Rebecca Patterson, Lorna Moussa, Kirsty Boyd

**Affiliations:** 1 172239 Usher Institute of Population Health Sciences and Informatics, The University of Edinburgh, Edinburgh, UK; 2 Scottish Partnership for Palliative Care, Edinburgh, UK

**Keywords:** Advance care planning, anticipatory care planning, advance directive, online health information, digital health, public involvement in research

## Abstract

**Introduction:**

Healthcare information is increasingly internet-based. Standards require websites to be ‘perceivable, operable, understandable and robust’ with relevant content for citizens in appropriate language. This study examined UK and international websites offering public healthcare information on advance care planning (ACP) using current recommendations for website accessibility and content and informed by a public engagement exercise.

**Methods:**

Google searches identified websites in English from health service providers, governmental or third sector organisations based in the UK and internationally. Target keywords that would be used by a member of the public informed the search terms. Data extraction used criterion-based assessment and web content analysis of the first two pages of each search result. Public patient representatives as key members of the multidisciplinary research team guided the development of the evaluation criteria.

**Results:**

A total of 1158 online searches identified 89 websites, reduced to 29 by inclusion/exclusion criteria. Most sites met international recommendations for knowledge/understanding about ACP. Differences in terminology, lack of information about ACP limitations and non-adherence to recommended reading levels, accessibility standards and translation options were apparent. Sites targeting members of the public used more positive, non-technical language than those for both professional and lay users.

**Conclusions:**

Some websites met accepted standards required to facilitate understanding and public engagement in ACP. Others could be improved significantly. Website providers have important roles and responsibilities in increasing people's understanding of their health conditions, future care options and ability to take an active role in planning for their health and care.

## Introduction

Digital health is an essential aspect of universal health coverage according to the World Health Organization (WHO) and includes the provision of high-quality, public health information for citizens.^1^ Research into online public health information campaigns found good uptake by digitally literate people via social media platforms and websites, achieving extensive coverage, consistently improving knowledge.^2^ Poor health literacy is associated with difficulties managing diverse health conditions, increased risk of long-term conditions, poorer health outcomes and higher mortality rates.^3^ Digital public information must address general barriers associated with poor health literacy alongside additional difficulties people can face when accessing and using online resources. As the majority of people accessing online healthcare resources are likely to be non-professionals, it is important to review such web-based tools despite them not, on the whole, being published in academic journals.^4^

Online sources must provide accurate, clear and appropriate materials that are comprehensible to most of the population.^5^ This directive is pertinent given one in five UK adults has impaired vision or hearing, cognitive impairment or learning disabilities, adding barriers to dissemination of health information online.^6^ In response to increasing use of digital information, the UK government Public Sector Accessibility Regulations (2018) require that all public information websites are ‘perceivable, operable, understandable and robust’. This means such websites must be reliable and easy for people to find, use and understand, with information presented using a simple structure and language.^7^

Advance Care Planning (ACP) as defined in a consensus exercise by the European Association for Palliative Care (EAPC) is a process that ‘enables individuals to identify their values, to reflect upon the meanings and consequences of serious illness scenarios, to define goals and preferences for future medical treatment and care, and to discuss these with family members and healthcare providers’.^8^ It is an internationally recognised approach to improving health outcomes for people with serious illnesses and life-limiting, long-term conditions. Consequently, ACP is relevant for people of any age who are at risk of progressive deterioration in health and/or dying.^9^ Taking part in an ACP process can help individuals and families feel better prepared for changes in their health. Shared decisions and agreed recommendations documented in an advance care plan or an advance care directive (ACD) can then help guide treatment and care decisions being made by the person's healthcare providers in the future.^10^

At the time of our study, little published research evaluated public-facing ACP information on UK or international websites guided primarily by user perspectives. Reviews of experiences during the COVID-19 pandemic suggested much of the available public information did not enable people to become better informed and engaged with ACP and recommended more person-centred approaches to online public awareness campaigns.^11,12^ This project aimed to address this gap with a research question based on the UK government standards: ‘What is the evidence to demonstrate public-facing ACP information websites are “perceivable, operable and understandable with a simple structure and language”?’^7^

## Methods

### Aim

We aimed to undertake a search for online materials in English that were most likely to be found by a member of the public searching for ‘advance care planning’ or a related specific term and then to conduct a qualitative web content analysis of the highest ranked search outputs to evaluate how well those sites met standards for online public health information and reflected the EAPC international consensus definition of ACP.

### Setting and search strategy

How members of the public perceive and use ACP websites was an essential component of this study, so we set up a public engagement process to guide our approach. The Policy and Public Communications lead for the Scottish Partnership for Palliative Care (RP) recruited volunteer citizens from across Scotland through existing public involvement networks to join a series of informal online discussion groups with the research team about their understanding of key terms and processes relating to ACP. COVID-19 precluded doing this in person. These volunteers included older people, relatives of care home residents, learning disability support workers, minority ethnic community leaders and advocates (n  =  33). We then identified four eligible Scottish ACP sites in an initial online search that were each reviewed by pairs of volunteers (n  =  20) invited from among the people who took part in the discussion groups and our two public and patient involvement (PPI) representatives (CA and LM) who are full members of the research team. The volunteers completed a ‘Google Forms’ questionnaire asking what they learned about ACP from each website, what they found useful, what was unhelpful and for any further comments on their experiences of accessing each website (Supplementary information 1). Insights from these engagement processes informed selection of the study search keywords and approach.

Following this public engagement exercise and in consultation with an information specialist and our public patient information representatives, we selected the following specific search terms: ‘advance care planning’, ‘anticipatory care planning’, ‘ACP’, ‘advance directive’, ‘ACD’ and ‘living will.’ The search terms were chosen to reflect keywords and topic names that members of the general public who had a reason to be looking specifically for information about ACP might use. This study covered Scotland where ‘anticipatory care planning’ encompasses people of all ages and those lacking decisional capacity, and that name has been adopted in preference to ‘advance care planning’. We also included specific names related to ACP processes such as ‘advance directive’ that are used widely internationally.

Websites from governmental, health service providers or third sector palliative care organisations based in Scotland, England, Northern Ireland or the Republic of Ireland were searched for initially, followed by international sites. Searches were all performed on the same university Windows PC using the Google search engine on the Chrome web browser in ‘incognito’ mode to prevent third-party internet cookies being created and attached to subsequent searches. This helped avoid re-direction to previously accessed websites.^13^ For each search, one of the specific ACP search terms was used in conjunction with the commonly used country name of one of the 193 United Nations member states. This reflected the fact that search engines usually use location and/or country of the user to prioritise the list of search results.^14^

Language was restricted to English to allow meaningful content analysis within available time and resources. Websites with commercial interests or that required a payment to access were excluded.^15^ Activity data give a measure of website popularity and visibility on search tools so we used SEMrush analytics software (www.semrush.com/) to examine website traffic volume by counting how many hits each site received over a 4-week period before the search.^16^ The sites were grouped accordingly as no data or low activity (under 100,000 hits), medium (100,000–1,000,000) or high (over 1,000,000 hits).

Final inclusion and exclusion criteria developed by the research team, including our two PPI representatives, are shown in Table 1. Decisions about which websites to include were made by the study researchers (AC and BM) supported by the project lead (KB) and PPI member (CA) based on these criteria and content relevance. SEMrush activity levels were reviewed but considered less important. In addition, the research team and a project steering group (expert members providing oversight of the project) provided lists of widely used public facing websites drawn from their clinical or other expert knowledge that were used to cross-check the reliability of the electronic searches.

**Table 1.** Inclusion and exclusion criteria applied to the website search strategy.

Inclusion Criteria	Exclusion Criteria
Freely available ACP website information.	ACP and website information requiring financial cost/subscription (public accessibility difficulties).
ACP website information targeted at members of the general public.	ACP website information targeted at healthcare professionals or finance/insurance professionals.
Information available in English.	Languages other than English.
Websites with a main focus on ACP in line with the EAPC international definition.	Websites with a main focus on other topics (i.e. euthanasia/assisted dying and health insurance).
Websites from reliable sources such as NHS providers, academic institutions and government agencies.	Websites with .com in their URL whose contents focused on corporate or commercial aspects of ACP (e.g. businesses and pharmaceutical companies).
Active sites assessed by SEMrush analytics software during the 4 weeks before the search date.	Websites returning nil/low site visits during the 4 weeks before the search date.

### Data extraction

Users searching via a web browser rarely go beyond the second page of any results list generated by an online search, and around 71.33% of individual searches result in a ‘click’ on the first page of website listings only.^17,18^ Search engines like Google display a variety of materials, videos and images on each page so the number of websites included will vary between searches. Data extraction was therefore limited to the websites displayed on first two ‘pages’ of each of the outputs of the Google ‘incognito’ searches carried out on the university PC used throughout the study. Details of each website meeting the inclusion criteria were charted using a Microsoft Word table cataloguing each website owner/organisation, country of origin, website Uniform Resource Locator (URL) and the date each site was accessed.

### Data analysis

Data analyses focused on three types of criteria for each website: ACP information; website accessibility in terms of display, navigation and readability; and emotional content. Web-based content analysis can consist of both the textual information available and the informational structure of a webpage. Quantitative and qualitative analyses are often integrated when synthesising data deductively in online content analyses.^19,20^ We applied qualitative methods to the textual information about ACP and simple, descriptive quantitative methods to the informational structure of the webpages selected.

For evaluation of ACP information provided on the websites, we drew on the 2017 EAPC consensus and its 12 recommendations about key elements of an ACP process along with the feedback from volunteers reviewing the test websites to guide decisions by the research team and public patient representatives on what information for patients and families would enhance their knowledge and understanding of ACP.^8^ We agreed on seven ACP information domains: clear explanation ACP terms/processes, aims of ACP, benefits of ACP, limitations of ACP, legal status of ACP, role of healthcare proxies and ACDs and how to make an ACP plan or ACD.

To analyse accessibility in terms of display, navigation and readability, we applied UK government accessibility regulations for public sector websites and mobile applications and combined them with recommendations from a review of strategies for evaluation of online public health information, including readability and usability.^6,7^ Display assessed seven aspects of visual presentation of website content that might aid public understanding of ACP. Firstly, text was assessed for font style and size in line with UK government standards for public health information as not all users will know how to change font size when visiting a webpage.^7^ This was important to our public volunteer reviewers who complained about small text they were not able to adjust on their device. Use of other media can increase engagement; therefore, images, videos, colour contrast and audio provisions were scored as present or absent. Each website was examined for third-party platforms, also known as social assets. These can increase dissemination of information and boost site profile, audience and attention. Finally, each website was checked for accessibility statements on the first page as these are designed to ensure maximum ease of interaction for users. For navigation, we recorded the presence and functionality of any hyperlinks or dropdown menus offered to provide additional information. Finally, we assessed readability using two well-established, complementary tools.^5,21^ The Flesch–Kincaid (FK) grading system rates reading level and measures how well the language used for website content suited the target audience (members of the general public), whilst the Simple Measure of Gobbledygook (SMOG) is an online readability assessment tool particularly designed for healthcare information that assesses use of words containing three or more syllables (www.webfx.com/tools/read-able/). Care was taken to comply with recommended procedures to ensure references, abbreviations, bullet points, hyperlinks, colons, semi-colons and hyphens were excluded from all retrieved texts to avoid underestimating reading scores due to perceived short sentences. In addition, we checked whether information was clearly available via a telephone helpline number for people who may have difficulty reading English and looked for a translation option for those unable to read or understand English.

Volunteers in our public engagement work emphasised that ACP is an emotive topic with the potential to cause distress and provoke strong emotions like fear, sadness or anger. Possible harms due to the emotional content of online health information was the final aspect we considered.^5^ Emotional content analyses (also known as sentiment analysis) can be inspected to determine if statements made on websites offer a positive, negative or neutral stance. Software tools automatically detect emotion, tone and urgency in online data and record the affect (positive, negative or neutral) which is called polarity. Such tools are used increasingly in palliative care research.^22,23^ For this study, we used a sentence level text and data mining (TDM) service available from the University of Edinburgh information services (https://digitext.edina.ac.uk/) to perform a series of emotional content analyses.^24^

Table 2 shows the three types of criteria and the assessment domains for each of them used in our evaluation of public-facing ACP websites. For the website data analyses, a social science researcher (AC) and one PPI team member who volunteers with a national cancer support charity (CA) examined each website independently based on these evaluation criteria. The two sets of assessments were each recorded on a Microsoft Excel spreadsheet and then discussed during online meetings whilst screen-sharing. Areas of agreement or conflict occurred in under 10% of cases, and a third reviewer (KB) aided consensus building during this decision-making process.

**Table 2.** Evaluation criteria for review of websites providing public information on advance care planning (ACP).

Mapping Criteria from UK Regulations, Expert Guidance, EAPC Definition and Public Feedback							
Criteria	Assessment Domains						
**1. Improved knowledge/understanding**	Clear explanation ACP terms/processes	Aims of ACP	Benefits of ACP	Limitations of ACP	Legal status of ACP	Role of POA*	How to make an ACP plan
**2a. Display accessibility**	Font and size	Use of images	Use of video	Use of colour contrasts	Use of audio	Social assets	Accessibility statement
**2b. Navigation accessibility**	Use of hyperlinks to other sources	Dropdown menu options					
**2c. Readability access**	Flesch–Kincaid score	SMOG score	Telephone helpline available	Translation options			
**3. Emotional content**	Tone rated with a polarity score						

*Power of attorney (legal proxy or substitute decision-maker).

## Results

**Figure 1.**
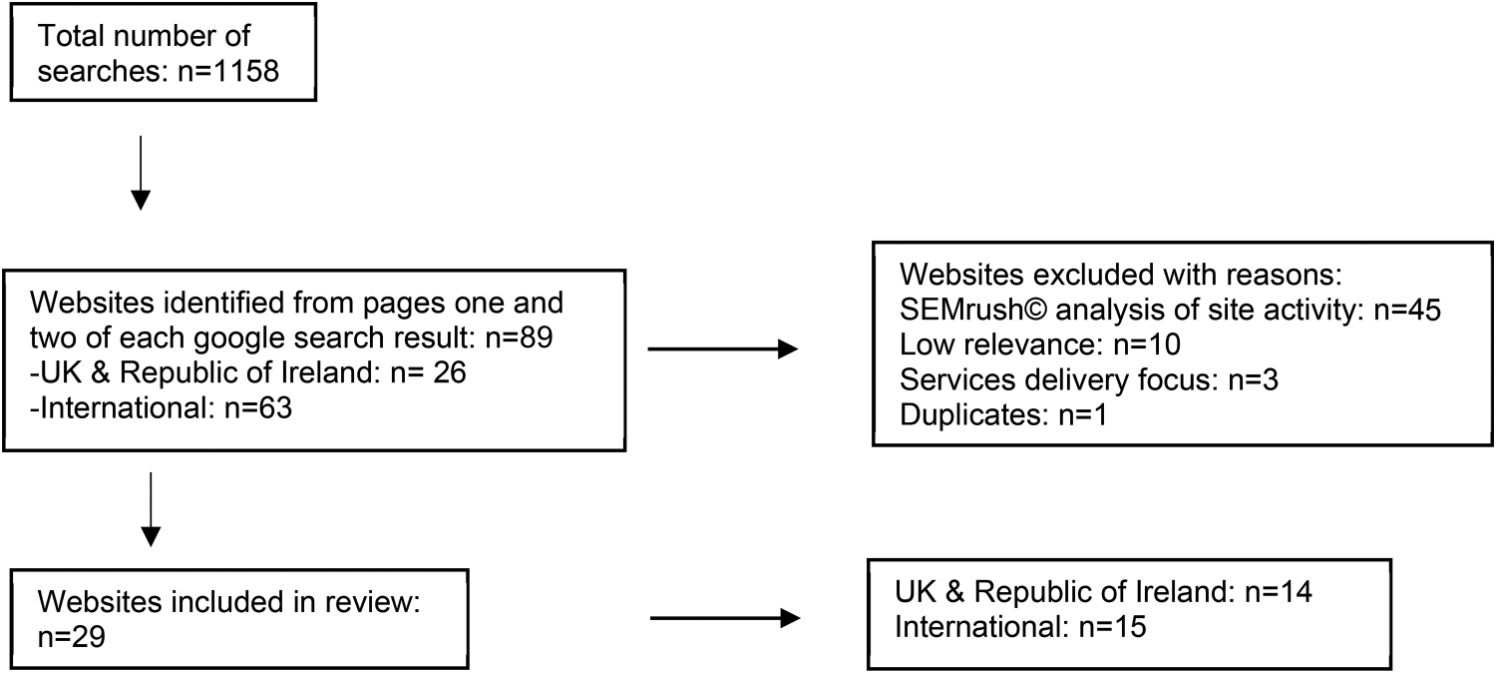
Flowchart of online ACP information search strategy.

A total of 1158 individual searches using Google incognito were performed over 4 days (17, 22 and 25 March 2021 and 28 April 2021). This resulted in initial identification of 89 websites with a main focus on ACP: UK and Republic of Ireland n  =  26 and international n  =  63. A total of 60 sites were excluded after review by the researchers based on the agreed criteria and content relevance about ACP information for members of the public (Figure 1). SEMrush activity data was of limited value with six sites that had low activity rates included (Table 3) and four with high rates excluded (Table 4). Three of the included low activity sites were recommended on the wider steering group list.^30,31,37^ A further 15 sites were removed due to low relevance (n  =  10), having a focus on service delivery (n  =  3) or duplicates (n  =  1).

**Table 3.** Websites that were included in the final review despite having ‘low’ numbers (0–100,000) of hits (see text for rationale).

ACP Websites Included with Low Hit Rates				
Location	Name of Site	Organisation	Date Accessed	SEMrush hits
UK/Republic of Ireland	Advance Care Planning/Advance Health care Directives	Irish Hospice Foundation, Republic of Ireland	17 Mar 21	4200
UK/Republic of Ireland	Advance Care Planning	Gold Standards Framework, England	22 Mar 21	12,700
UK/Republic of Ireland	Advance Care Planning	Compassion in Dying, England	22 Mar 21	19,900
UK/Republic of Ireland	Advanced (SIC) Care Planning	Myeloma UK, Scotland	25 Mar 21	33,800
UK/Republic of Ireland	Planning for the future	Scottish Partnership for Palliative Care, Scotland	22 Mar 21	1200
Australia	Advance Care Planning	Austin Health, Australia (funded by the Australian government)	17 Mar 21	17,500

**Table 4.** Websites that were excluded in the final review despite having ‘high’ numbers (over 1,000,000) of hits (see text for rationale).

ACP Websites Excluded with High Hit Rates				
Location	Name of Site	Organisation	Date Accessed	SEMrush hits
UK/Republic of Ireland	Advance Healthcare Directives	Irish Citizens Information, Republic of Ireland.	17 Mar 21	3,300,000
USA	Advance Care Planning Resources	Riverside Health System, VA, USA	22 Mar 21	1,000,000
USA	DNRs (Do Not Resuscitate) and Advance Planning for Kids: Everything you need to know	Support Organisation for Trisomy (SOFT), NY, USA	22 Mar 21	1,000,000
USA	Advance Health Care Directives and Living Wills	HelpGuide – international, non-profit organisation. CA, USA	22 Mar 21	5,800,000

As shown in Figure 1, 29 websites were included in the final review. These websites were divided into 2 groups: those identified as being run by organisations in the UK and Republic of Ireland, n  =  14 (Table 5), and international sites from other parts the world, n  =  15 (Table 6). Findings from the content analyses of the included websites for the evaluation criteria of ACP information and website accessibility are presented as pairs of tables (Tables 7 to 14) with UK/Republic of Ireland sites followed by international sites in each case.

**Table 5.** ACP public-facing websites: UK and Republic of Ireland.

Name of Site/Organisation	Origin	URL	Date Accessed
British Lung Foundation^25^	England	https://www.blf.org.uk/support-for-you/end-of-life/advance-care-plan	22 Mar 21
Cancer Research UK^26^	England	https://www.cancerresearchuk.org/about-cancer/coping/dying-with-cancer/making-plans/care-planning	22 Mar 21
Compassion in Dying^27^	England	https://compassionindying.org.uk/making-decisions-and-planning-your-care/planning-ahead/advance-care-planning/	22 Mar 21
Dementia UK^28^	England	https://www.dementiauk.org/get-support/legal-and-financial-information/advance-care-planning/	25 Mar 21
Edinburgh Health and Social Care Partnership^29^	Scotland	https://www.edinburghhsc.scot/longtermconditions/anticipatorycareplanning/	28 Apr 21
Gold Standards Framework^30^	England	https://www.goldstandardsframework.org.uk/advance-care-planning	22 Mar 21
Irish Hospice Foundation^31^	Ireland	https://hospicefoundation.ie/our-supports-services/policy-advocacy/advance-care-planning-ahds/	17 Mar 21
Macmillan Cancer Support^32^	England	https://www.macmillan.org.uk/coronavirus/healthcare-professionals/advance-care-planning	22 Mar 21
Myeloma UK^33^	England	https://www.myeloma.org.uk/news-and-stories/covid-19-information-hub/advanced-care-planning/	25 Mar 21
National Quality Improvement Team^34^	Ireland	https://www.hse.ie/eng/about/who/qid/other-quality-improvement-programmes/assisteddecisionmaking/advancehealthcaredirectivescodesofpractice.html	17 Mar 21
NHS Greater Glasgow and Clyde^35^	Scotland	https://www.nhsggc.org.uk/your-health/health-services/planning-care/planning-your-care/#	22 Mar 21
NHS Inform^36^	Scotland	https://www.nhsinform.scot/care-support-and-rights/palliative-care/planning-for-the-future/make-an-anticipatory-care-plan	22 Mar 21
Scottish Partnership for Palliative Care^37^	Scotland	https://www.goodlifedeathgrief.org.uk/content/advance_care_planning/	22 Mar 21
Social Care Institute for Excellence (SCIE)^38^	England	https://www.scie.org.uk/dementia/supporting-people-with-dementia/decisions/advance-care-planning.asp	22 Mar 21

**Table 6.** ACP public-facing websites: international.

Name of Site	Origin	URL	Date Accessed
American Cancer Society^39^	USA	https://www.cancer.org/treatment/finding-and-paying-for-treatment/understanding-financial-and-legal-matters/advance-directives/types-of-advance-health-care-directives.html	22 Mar 21
Austin Health (funded by Australian government)^40^	Australia	https://www.advancecareplanning.org.au/	17 Mar 21
British Columbia Health Link BC^41^	Canada	https://www.healthlinkbc.ca/more/health-features/planning-advanced-care	17 Mar 21
Cedars Sinai^42^	USA	https://www.cedars-sinai.org/patients-visitors/resources/advance-directive.html	22 Mar 21
Department of Health^43^	Australia	https://www.health.gov.au/health-topics/palliative-care/planning-your-palliative-care/advance-care-directive	25 Mar 21
Dementia Australia^44^	Australia	https://www.dementia.org.au/about-dementia/i-have-dementia/advance-care-planning	25 Mar 21
Dying with Dignity^45^	South Africa	https://dignitysouthafrica.org/why	22 Mar 21
Health Hub, Ministry of Health^46^	Singapore	https://www.healthhub.sg/a-z/medical-and-care-facilities/56/advance-care-planning	22 Mar 21
Health Navigator New Zealand^47^	New Zealand	https://www.healthnavigator.org.nz/health-a-z/a/advance-care-planning/	22 Mar 21
Ministry of Health Agency for Integrated Care^48^	Singapore	https://www.aic.sg/care-services/advance-care-planning	22 Mar 21
New Zealand Medical Association^49^	New Zealand	https://www.nzma.org.nz/resources/resources-for-patients/advance-directive/	22 Mar 21
Stanford School of Medicine^50^	USA	https://med.stanford.edu/palliative-care/patientsandfamilies/ACP.html	22 Mar 21
State of Israel Ministry of Health^51^	Israel	https://www.health.gov.il/English/Services/Citizen_Services/Pages/DyingPatientRequest.aspx	17 Mar 21
Sunnybrook Health Sciences Centre^52^	Canada	https://sunnybrook.ca/content/?page = advance-care-planning	17 Mar 21
Victoria Government Hub for Health Services^53^	Australia	https://www2.health.vic.gov.au/hospitals-and-health-services/patient-care/end-of-life-care/advance-care-planning/acp-overview	17 Mar 21

**Table 7.** Knowledge and understanding: UK and Republic of Ireland ACP websites.

	Clear Explanation of ACP	Aims of ACP	Benefits of ACP	Limitations of ACP	Legal Status of ACP Plan	Role of POA	How to Make an ACP Plan
British Lung Foundation	✓	✓	✓	x	✓	✓	x
Cancer Research UK	✓	x	✓	x	x	✓	x
Compassion in Dying, England	✓	✓	✓	x	✓	✓	✓
Dementia UK	✓	✓	✓	x	x	✓	✓
Edinburgh Health and Social Care Partnership	✓	✓	✓	x	x	x	x
Gold Standards Framework	✓	✓	✓	x	✓	✓	✓
Irish Hospice Foundation	✓	✓	✓	x	✓	✓	✓
Macmillan Cancer Support	✓	✓	✓	x	✓	✓	✓
Myeloma UK	✓	✓	✓	x	✓	x	✓
National Quality Improvement Team, Ireland	✓	✓	✓	x	✓	✓	✓
NHS Greater Glasgow and Clyde	✓	✓	✓	x	✓	✓	✓
NHS Inform, Scotland	✓	✓	✓	x	x	✓	✓
Scottish Partnership for Palliative Care (SPPC)	✓	✓	✓	x	x	✓	✓
Social Care Institute for Excellence (SCIE), England	✓	✓	✓	✓	✓	✓	✓

Item present: ✓; item absent: X.

ACP: advance care planning; POA: power of attorney.

**Table 8.** Knowledge and understanding: international ACP websites.

	Clear Explanation of ACP	Aims of ACP	Benefits of ACP	Limitations of ACP	Legal Status of ACP Plan	Role of POA	How to Make an ACP Plan
American Cancer Society	✓	✓	✓	x	✓	✓	✓
Austin Health (funded by Australian government)	✓	✓	✓	x	✓	✓	✓
British Columbia Health Link, BC	✓	✓	✓	x	✓	✓	✓
Cedars Sinai, USA	✓	✓	✓	x	✓	✓	✓
Department of Health Australia	✓	✓	✓	x	✓	✓	✓
Dementia Australia	✓	✓	✓	✓	✓	✓	✓
Dying with Dignity, South Africa	✓	✓	✓	x	✓	✓	✓
Health Hub, Ministry of Health, Singapore	✓	✓	x	x	✓	x	x
Health Navigator, New Zealand	✓	✓	✓	x	x	x	✓
Min of Health Agency for Integrated Care, Singapore	✓	✓	✓	x	x	✓	✓
New Zealand Medical Association	✓	✓	✓	✓	✓	✓	✓
Stanford School of Medicine, USA	✓	✓	✓	x	✓	✓	✓
State of Israel Ministry of Health, Israel	✓	✓	✓	x	✓	✓	✓
Sunnybrook Health Sciences Centre, Canada	✓	✓	✓	x	✓	✓	✓
Victoria Government Hub for Health Services, Australia	✓	✓	✓	✓	✓	✓	✓

Item present: ✓; item absent: X.

ACP: advance care planning; POA: power of attorney.

**Table 9.** Display attributes: UK and Republic of Ireland ACP websites.

Website	Font Size	Accessibility Statement (Font Size)	Social Assets	Use of Images	Use of Video	Use of Colour Contrast	Use of Audio
British Lung Foundation	Tahoma 11	X	5	✓	X	✓	X
Cancer Research UK	Arial 10.5	✓ (10.5)	2	X	X	✓	X
Compassion in Dying	Arial 16	✓ (11)	3	✓	X	✓	X
Dementia UK	Arial 15	X	4	X	X	✓	X
Edinburgh Health and Social Care Partnership	Roboto 10.5	X	0	✓	✓	✓	X
Gold Standards Framework	Arial 10.5	X	3	✓	✓	✓	X
Irish Hospice Foundation	Arial 11	X	3	✓	X	✓	X
Macmillan Cancer Support	Helvetica 11	✓ (9)	4	✓	✓	✓	X
Myeloma UK	Tondo 13.5	X	3	✓	X	✓	X
National Quality Improvement Team	Open Sans 10.5	✓ (10.5)	5	X	X	✓	X
NHS Greater Glasgow and Clyde	Nunito 11	✓ (10)	4	✓	X	✓	X
NHS Inform	Arial 12.5	✓ (12.5)	3	✓	✓	✓	✓
Scottish Partnership for Palliative Care	Arial 10.5	X	2	✓	✓	✓	X
Social Care Institute for Excellence (SCIE)	Arial 11	✓ (10.5)	4	✓	✓	✓	X

**Table 10.** Display attributes: international ACP websites.

Website	Font Size	Accessibility Statement (Font Size)	Social Assets	Use of Images	Use of Video	Use of Colour Contrast	Use of Audio
American Cancer Society	Source Sans Pro 13.5	(9)	3	✓	X	✓	X
Austin Health (funded by Australian Government)	Arial 13.5	✓ (13.5)	4	✓	✓	✓	X
British Columbia Health Link BC	Calibri 10.5	✓ (10.5)	4	✓	X	✓	X
Cedars Sinai, USA	Arial 13.5	✓ (10.5)	5	✓	X	✓	X
Department of Health Australia	Segoe UI 11	✓ (11)	2	X	X	✓	✓
Dementia Australia	Calibri 11	X	5	✓	✓	✓	✓
Dying with Dignity, South Africa	Arial 13.5	X	3	✓	X	✓	X
Health Hub, Ministry of Health, Singapore	Nunito 11	X	1	✓	X	✓	X
Health Navigator New Zealand	Source Sans Pro 10.5	✓ (10.5)	3	✓	✓	✓	X
Min of Health Agency for Integrated Care, Singapore	Arial 11	X	3	✓	✓	✓	X
New Zealand Medical Association	Source Sans Pro 11	✓ (10.5) via another link	3	X	X	✓	X
Stanford School of Medicine, USA	Source Sans Pro 11	X	3	✓	✓	✓	X
State of Israel Ministry of Health, Israel	Arial 11	X	2	✓	X	✓	X
Sunnybrook Health Sciences Centre, Canada	Open Sans 11.5	X	5	✓	✓	✓	X
Victoria Government Hub for Health Services, Australia	Helvetica 11	✓ (9)	5	X	✓	✓	✓

**Table 11.** Navigation: UK and Republic of Ireland ACP websites.

Website	Use of Hyperlinks to Other Sources	Dropdown Menu Options
British Lung Foundation	✓	X
Cancer Research UK	✓	X
Compassion in Dying	✓	X
Dementia UK	✓	X
Edinburgh Health and Social Care Partnership	✓	X
Gold Standards Framework, England	✓	X
Irish Hospice Foundation	✓	X
Macmillan Cancer Support UK	✓	X
Myeloma UK	✓	X
National Quality Improvement Team	✓	X
NHS Greater Glasgow and Clyde	✓	✓
NHS Inform, Scotland	✓	X
Scottish Partnership for Palliative Care	✓	X
Social Care Institute for Excellence (SCIE)	✓	X

**Table 12.** Navigation: international ACP websites.

Website	Use of Hyperlinks to Other Sources	Dropdown Menu Options
American Cancer Society	✓	X
Austin Health (funded by Australian Government)	✓	✓
British Columbia Health Link BC	✓	✓
Cedars Sinai, USA	✓	✓
Department of Health Australia	✓	X
Dementia Australia	✓	X
Dying with Dignity, South Africa	✓	✓
Health Hub, Ministry of Health, Singapore	✓	X
Health Navigator New Zealand	✓	✓
Min of Health Agency for Integrated Care, Singapore	✓	✓
New Zealand Medical Association	✓	✓
Stanford School of Medicine, USA	✓	✓
State of Israel Ministry of Health, Israel	✓	✓
Sunnybrook Health Sciences Centre, Canada	✓	✓
Victoria Government Hub for Health Services, Australia	✓	✓

**Table 13.** Readability attributes from UK and Republic of Ireland ACP websites.

Website	Flesch–Kincaid Grade^a^	SMOG Level (School Age)	Telephone Helpline Information Available	Translation Option
British Lung Foundation	9.5	10 (15–16)	✓	X
Cancer Research UK	6.7	8 (13–14)	✓	X
Compassion in Dying	10.8	11 (16–17)	X	X
Dementia UK	10.6	11 (16–17)	✓	X
Edinburgh Health and Social Care Partnership	10.1	11 (16–17)	✓	X
Gold Standards Framework, England	13.7	14 (19–20)	X	X
Irish Hospice Foundation	9.5	10 (15–16)	✓	X
Macmillan Cancer Support UK	8.5	10 (15–16)	✓	X
Myeloma UK	10.2	11 (16–17)	✓	X
National Quality Improvement Team	12.3	13 (18–19)	X	X
NHS Greater Glasgow and Clyde	8.3	9 (14–15)	X	✓
NHS Inform, Scotland	9	10 (15–16)	✓	✓
Scottish Partnership for Palliative Care	10.9	11 (16–17)	X	X
Social Care Institute for Excellence (SCIE)	9	10 (15–16)	X	X

^a^ Recommended standard FK grade: 8 or less.

**Table 14.** Readability attributes from international ACP websites.

Website	Flesch–Kincaid Grade Level	SMOG (Age)	Telephone Helpline Information Available	Translation Option
American Cancer Society	10.4	11 (16–17)	✓	✓
Austin Health (funded by Australian Government)	10	11 (16–17)	✓	X
British Columbia Health Link BC	9	10 (15–16)	✓	✓
Cedars Sinai, USA	10.5	12 (17–18)	✓	✓
Department of Health Australia	6.9	9 (14–15)	✓	✓
Dementia Australia	10.5	11 (16–17)	✓	X
Dying with Dignity, South Africa	13.8	15 (20–21)	X	X
Health Hub, Ministry of Health, Singapore	10.1	11 (16–17)	X	X
Health Navigator New Zealand	10.3	11 (17–17)	✓	✓
Min of Health Agency for Integrated Care, Singapore	9.1	10 (15–16)	✓	X
New Zealand Medical Association	12.4	13 (18–19)	X	X
Stanford School of Medicine, USA	7.1	8 (13–14)	X	✓
State of Israel Ministry of Health, Israel	17	17 (22–23)	X	✓
Sunnybrook Health Sciences Centre, Canada	7.9	9 (14–15)	X	X
Victoria Government Hub for Health Services, Australia	12.7	14 (19–20)	X	X

### Criterion 1: information to improve knowledge and understanding of ACP

All 29 websites provided information to enhance knowledge and understanding among members of the public as a primary purpose (Tables 7 and 8). The sites all incorporated public information content in line with the EAPC guidance. UK/Republic of Ireland and international websites referred to ACP and explained it appropriately. The four Scottish websites all described the broader scope of ‘anticipatory’ care planning.^29,35,36,37^ Two websites from the Republic of Ireland used the term ‘Advance Healthcare Directives’.^31,34^ Other terms from sites included ‘Advance Healthcare Planning’,^50^ ‘Advance Medical Directives’,^51^ ‘Advanced Statement’^38, 39^ and ‘Living Will’.^45^ The Ministry of Health, Singapore, referred to ‘Elder Care Planning’ for ACP giving the impression that ACP is only for older people.^48^ Two sites from the USA used the term ACD interchangeably with ACP^42,50^ as did two from Australia.^43,44^ All websites provided a description of the aims and benefits of ACP for patients and family caregivers such as having greater autonomy and choice, but only four gave any information about the limitations associated with inherent future uncertainties of many illness trajectories and evolving treatment options.^34,44,49,53^ An example from the New Zealand Medical Association presented advantages and disadvantages together:

While the Medical Association recognises the advantages of advance statements in terms of encouraging openness, dialogue and forward planning, it also draws attention to potential disadvantages. Health professionals and the public should be aware that treatment decisions are complex and practice is constantly evolving. If advance directives are made a long time before capacity is lost, treatment options may have significantly changed. Over time, patients’ views can also change about what constitutes a tolerable existence. Advance directives cannot encompass unforeseen possibilities and options. Therefore, while upholding patients’ rights to decide in advance, the NZMA also emphasises that patients need to think carefully about the risks associated with committing themselves in advance.^49^

Information about the legal status of an ACP plan or an ACD was not offered routinely and often difficult to locate. A South African website provided an explanation of ACD legal status, but this was in an appendix showing their advance directive/living will planning guide.^45^ The various titles for a legal proxy (or substitute decision-maker) appointed through a power of attorney (POA) process to make health and care decisions on behalf of a person lacking capacity reflected the different legislative systems of countries represented in the review. This was most notable for the UK and Republic of Ireland where organisations based in England and Wales referred to ‘Lasting Power of Attorney’,^25,26,27,38^ whilst sites in Scotland used ‘Welfare Power of Attorney’^35^ and in Ireland ‘Enduring Power of Attorney’.^31,34^ Even if ‘Power of Attorney’ was mentioned on the first two pages of a site, finding relevant information about what a POA meant and its legal status could require detailed searching and navigational skills including the use of external links to other sites.^32,44^ However, two UK national websites did offer clear POA sections for people living in Scotland, England, Wales and Northern Ireland.^27,28^ One national charity website which included details for all four nations covered Northern Ireland in a different section to the other devolved UK nations, using an ‘Advance Decision to Treat’ link.^32^ From an international perspective, the term ‘Durable Power of Attorney’ was used in South Africa^45^ and commonly incorporated into information about Advance Care Directives in the USA and Canada,^39,41,42,50^ although one Canadian website adopted the term ‘Continuing Power of Attorney’.^52^ ‘Enduring Power of Attorney’ was used widely in Australia and New Zealand,^40,44,47,49,53^ but one Australian website used the expression ‘Substitute Decision-maker’.^43^ Two Singapore sites used ‘Lasting Power of Attorney’,^46,48^ and another just referred to ‘Power of Attorney’.^51^ Most websites described how to make an ACP plan and/or an ACD although this was absent from three UK/Republic of Ireland and one international website.

### Criterion 2a: display

UK government accessibility guidelines recommend the use of a ‘Sans Serif’ font such as Arial or Helvetica. A minimum size of 12 points should be applied and at least a 16-point option for visually impaired people. As shown in Tables 9 and 10, a majority of the sites (n  =  21) used a font size below recommendations with just 4/14 (UK/Republic of Ireland) and 4/15 (international) websites complying. Three quarters of UK and Republic of Ireland websites (11/14) used images to communicate ACP information as did most international websites (12/15). Videos were included on 6/14 UK and Ireland websites and 7/15 for international sites, but some video links did not work.^44,50^ All websites included colour contrast to aid user experience and understanding and colour-blind–friendly options. Audio communication was least common occurring on one Scottish site^36^ and three Australian sites.^43,44,53^ This facility allows a user to click a link, normally located at the top of a website page, and listen to an audio recording of scripted pages.

Those websites displaying accessibility statements (7/14 UK and Republic of Ireland and 7/15 international) to direct users to alternative means of communication (such as guidance on how to use accessibility functions) had information and links at the bottom of each homepage. However, these were sometimes difficult to see. One UK site chose accessible language ‘help using the website’,^38^ and an international site used the term ‘Non-discrimination’.^42^ Smaller print was noted on almost all these accessibilities links with just one Australian site^40^ above the recommended 12-point font size. One Scottish^37^ and four international sites^46,47,48,50^ included a ‘triple A’ icon at the top of each page to indicate an option to increase the font size automatically.

### Criterion 2b: navigation

All 29 websites provided hyperlinks to resources within their website or links to external platforms as navigation tools (Tables 11 and 12). Dropdown menus for additional information options were more common on international sites (11/15) but found in just one UK site (1/14).

### Criterion 2c: readability

Readability measured first with FK grading found just one UK/Republic of Ireland^26^ and three international websites^44,50,52^ which adhered to the recommended readability standard of FK grade 8 or under (Tables 13 and 14). A further two UK websites^32,35^ scored FK grade of 8.3–8.5 and could be considered adequate. SMOG levels confirmed the FK scores with 5 sites overall rated under 10 and representing an educational level up to 15 years.

Of the UK/Republic of Ireland sites, 8/14 had a direct telephone helpline number on their homepage as did 8/15 international sites. Two other sites included telephone contact details through additional links or on their website page footers.^36,44^ Only two of the UK/Republic of Ireland websites provided readily accessible translation options.^35,36^ The international websites offered more such services (7/15). For example, the Department of Health Australia offered content in 26 languages via an electronic link and a contact telephone number for further assistance.^43^ Cedars Sinai in the USA offered 15 languages plus a contact number for interpreter services.^42^ Similarly, Health Navigator New Zealand provided four language translations and interpreter services.^47^ The American Cancer Society offered 14 languages,^39^ British Columbia Health Link 9,^41^ and State of Israel Ministry of Health 6,^51^ and Stanford, USA, had a Spanish translation.^50^

### Criterion 3: emotional content

**Figure 2.**
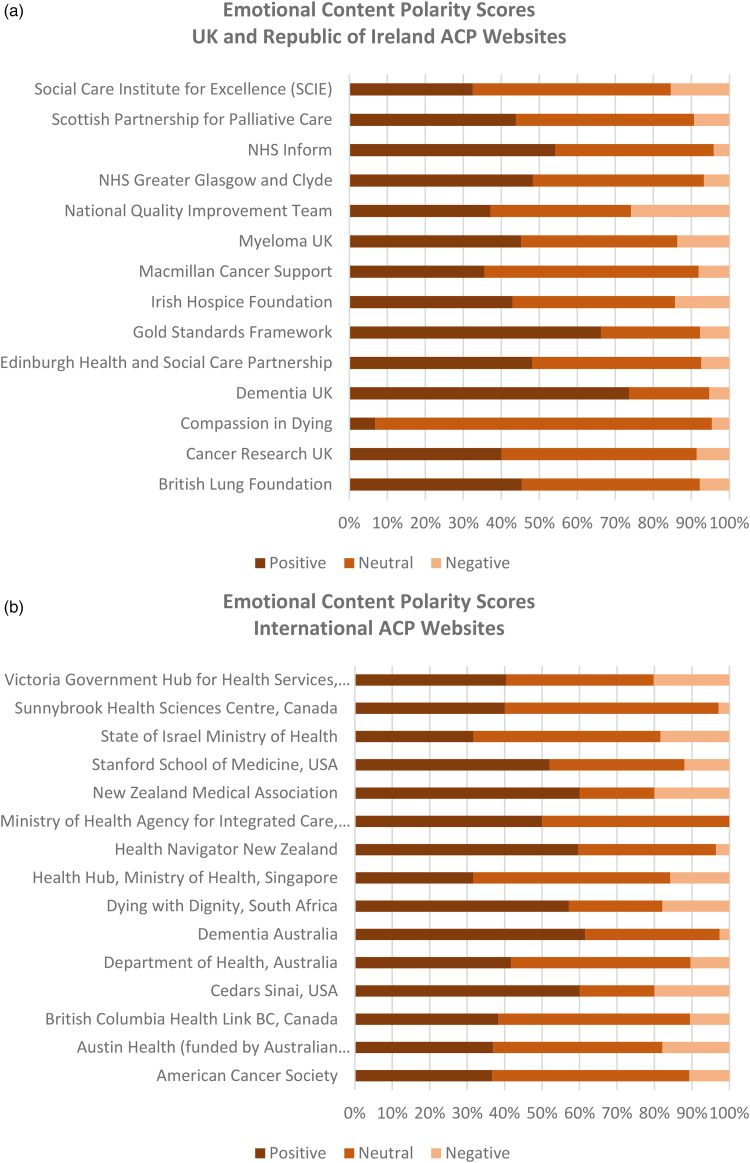
(a) Emotional content: UK and Republic of Ireland ACP websites. (b) Emotional content: international ACP websites.

Emotional content analyses showed a wide range of scores, although all 29 websites had more positive than negative polarity scores (Figure 2(a) and (b)). Those sites that scored higher in positive polarity introduced ACP (or its equivalent) using relatively simple language with fewer formal concepts. The following examples from National Health Service (NHS) Inform, Scotland, and Cedars Sinai, USA, show language giving higher positive scores for emotional content when introducing ACP on the homepage of a website.

Anticipatory Care Planning (ACP) is where you talk about what matters most when making plans for your care in the future. You can talk about this with those close to you, and your doctor, nurse or care worker. Your care team want to know what's important when they're planning treatment and care with you.^36^

An Advance Healthcare Directive allows you to share with your physicians, nurses and others taking care of you, including family and friends, what is most important to you when thinking about your health, healthcare and illness. The process of completing an Advance Healthcare Directive is also important because it can help you talk with loved ones about these things.^42^

Websites with more negative polarity scores tended to introduce ACP with formal or professional language and concepts. Examples from the State of Israel Ministry of Health and Victoria Government Hub for Health Australia show how such sites introduced ACP:

The law permits any citizen over the age of 17, who is fit to make decisions, to provide preliminary medical directives or to appoint a power of attorney, for the event in which they will become a dying patient who is not fit to make decisions.^51^

Advance care planning allows people to clearly express their values and preferences to inform clinical decision-making when they are unable to directly participate. ….. An Advance Care Directive is the only legally recognised document that a person can record their medical treatment preferences in. However, should a person lose decision-making capacity, any written record of their values or medical preferences must be considered by their medical treatment decision maker.^53^

When exploring website content in more depth, the presentation style tended to be consistently informal or more formal. Two examples of accessible language with positive emotional content that encouraged people to talk with family, friends and their own healthcare team about ACP are shown. One was a further excerpt taken from Cedars Sinai, USA, and another from Health Navigator, New Zealand:

STEP 1: Talk to Your Loved Ones. This is an important step. Your family members and close friends may help in your decision-making process. Remember, you are the expert about what matters most to you, and it's best to share this information with your loved ones in advance of any unforeseen need.^42^

If you have a plan written down, make sure you share it with your family/whānau and your healthcare team and anyone else you would like to share it with. It is important your whānau and other loved ones know you have a plan and where the plan is kept. Or you can give them a copy.^47^

By contrast, an extract from the National Quality Improvement Team, Republic of Ireland, site exemplifies formal language, use of the third person and negative tone not well suited to members of the public:

A person cannot include requests in their Advance Healthcare Directive which are criminal acts in law including euthanasia or assisted suicide. An Advance Healthcare Directives Multidisciplinary Working Group has been established under the Act by the Minister for Health to prepare draft codes of practice in relation to the Advance Healthcare Directive provisions contained in the Act.^34^

## Discussion

We examined online information about ACP aimed at members of the public from 29 websites. The search process to determine which sites to choose and evaluation criteria to guide us in assessing the key elements on which to focus was guided by volunteer citizens and our public patient research team members, one of whom reviewed all the included websites alongside a social science researcher. Despite efforts to build international consensus around definitions and intended outcomes of ACP, our findings showed variable terminology and descriptions adopted by different websites within the UK and internationally.

ACP remains a core aspect of healthcare planning for people with serious illnesses in the UK and internationally with a recent review confirming the benefits of holistic ACP processes.^54^ However, public engagement and awareness of ACP is still low. Less than 5% of patients presenting to hospital with an acute medical emergency in England had an ACP available to guide care.^55^ A review of ACP among hospitalised older adults showed similarly low ACP numbers completing an ACP process.^56^ ACP is recognised to be a complex, multidimensional process involving patients, family members and proxy (surrogate) decision-makers where public information to support engagement and involvement play an important role.^57,58^ People will use the internet increasingly to search for and access such healthcare information for ACP; therefore, resources need to be relevant and accessible to a wide range of citizens.^11,59^ Our use of academic researchers combined with members of the public helps indicate which elements may be most important to prioritise in developing materials that meet the needs of their potential users. Previous research has suggested that providers of online ACP information can do more to stress the importance of meaningful conversations, frame ACP as an iterative process and move away from a focus on documenting plans: something that our findings support.^12,20^

Our search methodology was informed by a recently published catalogue of ACP and end-of-life care resources.^59^ We evaluated whether websites likely to be found by patients and families met three types of criteria: ACP information reflecting international consensus definitions, website accessibility in line with governmental standards for public sector websites and emotional content given that ACP relates to sensitive topics around death and dying. This may reflect continued debate among experts in the field,^60^ but can be confusing for members of the public. Few sites gave balanced information about the inherent uncertainties of different illness trajectories and limitations of ACP^61^ nor went beyond detailing the status of proxy decision-makers to discuss the known challenges they face, another important limitation recognised increasingly by ACP programmes.^62,63,64^

In terms of site design and accessibility, over 75% of both UK/Republic of Ireland and international websites we reviewed did not meet the recommended reading level for public information, thereby potentially excluding a substantial proportion of the general public. In addition, font size was well below standard in the majority of all the websites. Websites that included accessibility statements often used smaller than recommended font sizes for those statements. Some websites incorporated a function to increase font size on their homepage, but inexperienced users may not know what this icon means. Small font size reduces accessibility in public-facing health websites, adversely impacting web users with reading difficulties or limited health literacy skills.^65,66^ All websites included hyperlinks but some were inoperative and triggered error messages. Video material and hyperlinks are prone to disruption and require regular review to keep content and technical aspects current and operational.^67^ Almost all websites provided a contact or helpline telephone number to support users, but these were not always adequately displayed on homepages again restricting access. Translation options were provided to a much greater extent in international sites than for the UK/Republic of Ireland, another important equity deficit.^68,69^ Two recent studies of the growing number of publicly available interactive online tools to support ACP found both quality of the content and readability varied substantially.^4,59^ Surveys of how caregivers used digital technologies and online information during COVID-19 highlighted the importance of developing digital support services particularly for older people and marginalised groups along with credible information from professional and governmental organisations.^70^

Healthcare websites should meet high accessibility standards suitable to all people, including accuracy and completeness; clear layout, graphics and images; ease of navigation; readability; accessibility for people with disabilities; translation options; and authorship disclosures.^71^

Analysis of emotional content showed that many organisations used appropriate language, avoiding technical words or concepts with the potential to provoke anxiety or distress among lay users. Websites which targeted a range of audiences tended to adopt more formal, complicated descriptions, making their content less engaging and comprehensible. For the potential benefits of ACP for more patients and families to be realised, online public information must help address the cognitive and emotional barriers that prevent people from considering the option of planning ahead for changes in health with their family and healthcare team.^72^

Our evaluation identified several websites adhering to good practice guidelines for online ACP information that are well placed to help people gain knowledge of their health conditions and possible future changes engage in conversations with family members and work collaboratively with clinicians and other healthcare providers when discussing a meaningful, individual ACP plan. Our use of public engagement exercises and public patient representatives to guide the evaluation indicates the importance of involving such expertise in the design and development of online ACP materials. Future research and improvement science studies should adopt a broad range of participatory mixed methods and bring together expertise in online education, behavioural science and co-design.^73^

## Limitations

The study is limited in that search results cannot be replicated exactly due to the continually evolving algorithms operated by search engines which alter website positions on results pages. In addition, the included sites may well have been updated since our study. We restricted our evaluation to websites with a primary purpose of providing public information about ACP whilst being aware of the growing number of interactive websites supporting participation in ACP that have been reviewed by others. Findings were based on websites where English is the primary language, and excluding websites in other languages and sites for lower income countries is a limitation we could not address due to resource constraints.

## Conclusions

Some websites evaluated well, incorporating many recommended functions and features to facilitate public understanding and engagement in ACP. Other websites were deficient in basic attributes such as readability level and functionality. Terminology and descriptions in websites that target a range of audiences may be unhelpful and could discourage members of the public from becoming better informed and empowered to take an active role in managing their future health and care planning. Public health communicators and website designers can do more to adapt their content and address user-centred needs including accessibility and languages across diverse populations, cultures and minority groups.

## Supplemental Material

sj-pdf-1-dhj-10.1177_20552076231180438 - Supplemental material for Online public information about advance care planning: An evaluation of UK and international websites Click here for additional data file. Supplemental material, sj-pdf-1-dhj-10.1177_20552076231180438 for Online public information about advance care planning: An evaluation of UK and international websites by Anne Canny, Bruce Mason, Clare Atkins, Rebecca Patterson, Lorna Moussa and Kirsty Boyd in DIGITAL HEALTH
