# Supplementary material for: Online public information about advance care planning: An evaluation of UK and international websites
Source: Digit Health. 2023 Jun 22;9:20552076231180438. doi: 10.1177/20552076231180438 (PMC10291539; doi:10.1177/20552076231180438)
Supplement: sj-pdf-1-dhj-10.1177_20552076231180438 - Supplemental material for Online public information about advance care planning: An evaluation of UK and international websites [file sj-pdf-1-dhj-10.1177_20552076231180438.pdf]

4-Actions Care Planning Study

THE UNIVERSITY of EDINBURGH | usher institute

ACP Website Review Answer Sheet

Name of Website Under Review:
NHS Inform - Make an anticipatory care plan:
https://www.nhsinform.scot/care-support-and-rights/palliative-care/planning-for-the-future/make-an-anticipatory-care-plan

1. 1. What did you find out about anticipatory care planning from this website?

2. 2. What was helpful on this website for giving you information about anticipatory care planning in Scotland?

3. 3. What was unclear or not helpful on this website?

4. 4. Please add any other comments you may have about the website or how it could be improved.

Thank you for taking time to answer our questions. Your feedback is valuable.
additional text here

This content is neither created nor endorsed by Google.

Google Forms
